# Advances in elucidating the function of leucine-rich repeat protein kinase-2 in normal cells and Parkinson's disease

**DOI:** 10.1016/j.ceb.2020.01.001

**Published:** 2020-04

**Authors:** Matthew Taylor, Dario R. Alessi

**Affiliations:** Medical Research Council Protein Phosphorylation and Ubiquitylation Unit, School of Life Sciences, University of Dundee, Dow Street, Dundee, DD1 5EH, UK

**Keywords:** Rab GTPase, RILPL1, Signal transduction, Protein kinase, Ciliogenesis, Lysosomal stress, Neuroinflammation

## Abstract

Autosomal dominant missense mutations that hyperactivate the leucine-rich repeat protein kinase-2 (LRRK2) are a common cause of inherited Parkinson's disease and therapeutic efficacy of LRRK2 inhibitors is being tested in clinical trials. In this review, we discuss the nuts and bolts of our current understanding of how the LRRK2 is misregulated by mutations and how pathway activity is affected by LRRK2 binding to membrane, microtubule filaments, and 14-3-3, as well as by upstream components such as Rab29 and VPS35. We discuss recent work that points toward a subset of Rab proteins comprising key physiological substrates that bind new sets of effectors, such as RILPL1/2, JIP3 and JIP4 after phosphorylation by LRRK2. We explore what is known about how LRRK2 regulates ciliogenesis, the endosomal–lysosomal system, immune responses and interplay with alpha-synuclein and tau and how this might be linked to Parkinson's' disease.

## Introduction

The clinical genetics of leucine-rich repeat protein kinase-2 (LRRK2) can be traced back to 1978, when researchers described a family in the Sagamihara City, Japan, in which at least 5 generations were afflicted with autosomal dominant inherited Parkinson's disease (PD), resembling idiopathic disease with age of onset typically after 50 years [1]. Linkage analysis located the PD causing mutation to a region within chromosome 12 termed PARK8 [2]. Contemporaneous work by two groups in 2004 pinpointed the disease-causing mutations to the gene encoding LRRK2 [3,4]. All members of the Sagamihara family affected with PD exhibited a heterozygous I2020T missense mutation within the LRRK2 protein kinase domain [5]. It was soon realized that LRRK2 mutations are the most frequent cause of inherited PD, accounting for at least 5% of familial and 1–2% of idiopathic PD [6].

PD affects an estimated 7 million people worldwide and all attempts to slow the progression of Parkinson's have thus far failed [7]. The cardinal symptoms, shaking, rigidity, and slowness of movement arise from degeneration of dopaminergic neurons located within the substantia nigra [8]. Dementia and behavioral disorders are also common in the advanced stages of the disease [8].

## LRRK2 domain structure and impact of pathogenic mutations

**Figure 1:**
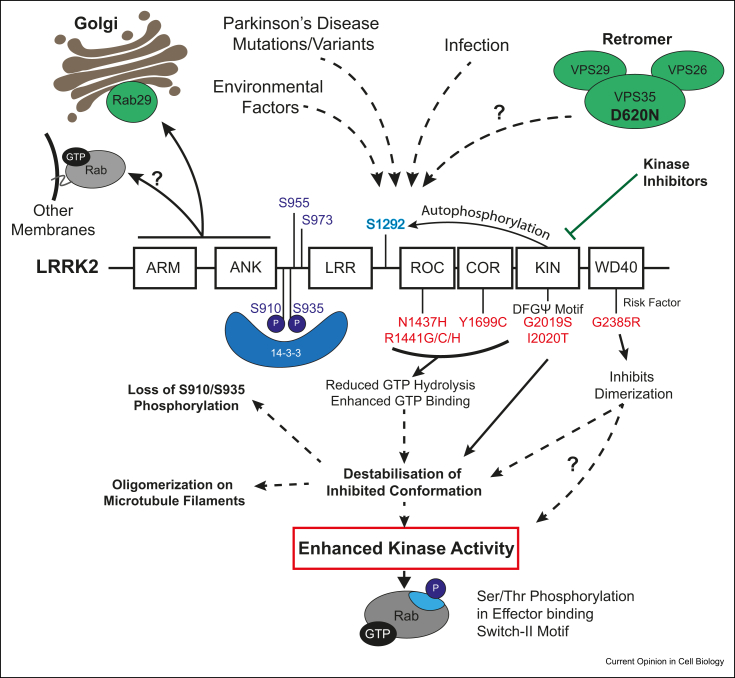
**LRRK2 structure, upstream regulation and pathogenic mutations.** Schematic of LRRK2 domain structure. ARM, Armadillo; ANK, Ankyrin; LRR, Leucine-rich repeat; ROC, Ras of complex; COR, C-terminal of ROC; KIN, Kinase. Depicted are the clear-cut autophosphorylation site S1292 (Blue) and other phosphorylation sites S910, S935, S955, S973 (Purple) with S910/S935 mediating 14-3-3 binding. Familial Parkinson's disease pathogenic mutations (Red) indicated with the proposed pathological mechanism enhancing kinase activity. Upstream regulation of LRRK2 shown previously, including Rab29 recruitment to Golgi, possible LRRK2 recruitment by unknown Rabs (gray) to other organelle membranes or vesicles and the pathogenic VPS35[D620N] mutation. Dashed Lines indicate more-than-one step processes. LRRK2, leucine-rich repeat protein kinase-2.

LRRK2 is a large multidomain enzyme bearing tandem Roco type GTPase and catalytic kinase domains, in addition to armadillo, ankyrin and leucine-rich repeats, as well as a C-terminal WD40 domain (Figure 1). LRRK2 exists predominantly as a dimer that likely represents the active species [9, 10, 11]. It is expressed most highly in immune cells (neutrophils, monocytes, and B cells), lung, kidney, and intestine, with significantly lower expression levels in brain [12●,^13^]. The Roco domain comprises a Ras-like GTPase fold named Roc (Ras of complex) followed by a dimerization fold termed Cor (C-terminal of Roc). Roco domains are found in diverse species including bacteria and plants [14]. Mammals possess 4 proteins bearing a Roco domain: 3 kinases (LRRK1, LRRK2, and DAPK) and a scaffolding protein (MASL1). Roco GTPases bind GTP/GDP with low micromolar affinity and may therefore not necessitate a guanine nucleotide exchange factor (GEF) or a GTPase activating proteins (GAP) enzyme to exchange nucleotides [15]. GTP binding is essential for kinase activity as mutations that abolish GTP binding to the ROC domain (T1348N) ablate LRRK2 kinase activity [16,17●].

Seven well-characterized, pathogenic, and dominantly inherited missense mutations are located within the Roc (N1437H, R1441G/C/H), Cor (Y1699C), and kinase (G2019S, I2020T) domains of LRRK2 [18] (Figure 1). Substitution of serine for glycine at position 2019 in the human LRRK2 protein (G2019S) is the most common mutation. In addition, a LRRK2 variant located within the WD40 domain (G2385R), that is relatively common in Chinese Taiwanese populations moderately increases PD risk [19].

The pathogenic mutations within the LRRK2 kinase domain (G2019S and I2020T) enhance LRRK2 activity moderately, under 2-fold *in vitro* [20, 21, 22] and *in vivo* as judged by phosphorylation of Rab substrates [23]. Autophosphorylation of LRRK2 at Ser1292 is enhanced up to 4-fold by the G2019S mutation [24]. The G2019S and I2020T residues lie within the DFGψ (ψ = aliphatic side chain I,L,V,M) motif found in all kinases that coordinates a hydrophobic spine structure controlling catalytic activity [25●]. In LRRK2, this motif possesses the sequence DYGI (residues 2017–2020). The Tyr2018 residue within this motif is proposed to form a hydrogen bond that serves as a ‘brake’ on kinase activity by stabilizing LRRK2 in a less active conformation [25●], a finding that has been confirmed in recent structural work [26●●]. The G2019S and I2020T mutations are thus thought to enhance LRRK2 catalytic activity by disrupting the inhibited kinase domain conformation [25●].

The LRRK2 Roc and Cor domain mutations (N1437H, R1441G/C/H,Y1699C) suppress GTPase activity and promote GTP binding [27]. Enhanced GTP binding is thought to mediate the 3–4-fold increase in kinase activity observed toward Rab substrates and autophosphorylation at Ser1292 observed *in vivo* [23,24]. The G2385R mutation disrupts dimerization of the WD40 domain, slightly enhancing LRRK2 activity *in vivo* through an unknown mechanism [28●] (Figure 1).

LRRK2 possesses multiple phosphorylated 14-3-3 binding residues, including Ser910 and Ser935 sites [29]. Several PD-associated mutants (R1441 C/G, Y1699C, I2020T) suppress Ser910 and Ser935 phosphorylation and hence reduced 14-3-3 binding. When overexpressed, these mutants are recruited onto the microtubule network [30] and LRRK2 association occurs in a well-ordered, oligomeric periodic manner [31]. Wild type LRRK2 or even the G2019S mutant does not associate well with microtubules [25●]. However, inhibitors that bind to the ATP site, termed type I, in a manner proposed to destabilize the inhibited kinase conformation of LRRK2, promote recruitment of wild type and G2019S LRRK2 to microfilaments [25●]. A 14 Å *in situ* cryo-electron tomography structure of the LRRK2[I2020T] bound to microtubules indicates that LRRK2 oligomerizes as a right-handed double-helix around microtubules, which are left-handed [32●●]. The GTPase domain points toward the microtubule polymer, suggesting that the GTPase cycle could regulate microtubule binding; the kinase domain is instead exposed to the cytoplasm, perhaps poised to phosphorylate substrates. The dimeric doughnut-shaped WD40 fold bridges two protomers together, with the G2385R mutation abolishing microfilament association [32●●]. This structure reveals a second undefined oligomerization interface mediated by non-catalytic domains [32●●]. The *in situ* cryo-electron tomography structure of the LRRK2[I2020T] differs significantly from a model of full-length LRRK2 in solution, not bound to microtubules, obtained using a combination of chemical cross-linking, negative-stain EM, and small-angle X-ray scattering [33].

Recent work has revealed a 3.5 Å cryo-EM structure of the catalytic region of LRRK2 encompassing the ROC-COR-kinase and WD40 domains, in which the LRRK2 kinase domain is in the inactive ‘open’ conformation and Roc domain complexed to GDP [26●●]. Striking features of the LRRK2 structure demonstrate major interactions between the kinase domain and the GTPase domain, suggesting how conformational changes induced by GTP or GDP binding and hydrolysis might be coupled to kinase domain conformation [26●●]. The extreme C-terminal 28 amino acids of LRRK2 form an extended α-helix that binds along the entire kinase domain, interacting with both its C- and N-lobes. Interactors or possible phosphorylation sites located within this C-terminal region could readily impact on kinase activity [26●●]. Integrating data with the 14 Å cryo-electron tomography *in situ* structure [32●●] suggests that the LRR region wraps around the N-lobe of the kinase domain, placing the known S1292 autophosphorylation site [24] in close proximity to the kinase's active site [26●●]. Further modeling and experimental studies suggest that the LRRK2 kinase domain needs to adopt the closed conformation to interact with microtubules [26●●]. Type I inhibitors that induce the closed kinase conformation, such as MLi-2, promote microtubule binding and inhibit kinesin and dynein motility *in vitro* by causing a ”'roadblock'” for motor proteins [26●●]. This is reversed both *in vitro* and *in vivo* by adding type II inhibitors, such as GZD-824, that promote the open conformation of the LRRK2 kinase domain [26●●]. In future work, it would be interesting to investigate whether microtubule-associated LRRK2 phosphorylates stalled motor proteins bound to cargo-associated Rab GTPases and if so whether this leads to cargo release. It will be also interesting to compare the downstream biological effects of inhibitors that trap LRRK2 in the microtubule binding closed conformation versus the nonbinding open conformation. As the wild type, G2019S and G2385R mutants that do not interact with microtubule filaments are still active and phosphorylate Rabs *in vivo*, the role that microtubules play in regulating LRRK2 and Rab phosphorylation requires further analysis. Whether endogenous LRRK2 oligomerizes with microtubule filaments also needs to be determined.

All LRRK2 type I inhibitors induce dephosphorylation of Ser910 and Ser935 residues [34] and a cluster of other close-by sites [35], which has been exploited to probe *in vivo* efficacy of LRRK2 inhibitors. It is not understood how LRRK2 activity or destabilization of the inactive conformation of the LRRK2 kinase domain is linked to Ser910/Ser935 phosphorylation and whether these sites are regulated via a complex autophosphorylation mechanism or controlled through the activity of a protein phosphatase-1 complex [17●,^36^]. How type II inhibitors impact LRRK2 phosphorylation has not been tested.

## LRRK2 inhibitors as potential therapeutic agents for PD

As pathogenic mutations increase kinase activity, LRRK2 inhibitors might offer therapeutic benefit for treating or preventing LRRK2-driven PD [37,38]. Elevated LRRK2 activity has also been observed in idiopathic PD, suggesting that inhibitors may benefit patients beyond those carrying LRRK2 mutations [39●●]. Pharmaceutical companies have developed LRRK2 inhibitors for treatment and prevention of PD and clinical trials have commenced and/or are planned (see https://clinicaltrials.gov). Studies with LRRK2-deficient rodents [40] and nonhuman primates treated with inhibitors have highlighted potential concerns in lung (abnormal accumulation of lamellar bodies in type 2 pneumocytes) and kidney (hyaline droplets and a lipofuscin-like brown pigment in the renal proximal tubular epithelium) [41]. These findings generally phenocopy results obtained in LRRK2-deficient mouse [42,43] and rat studies [40], indicating that they are on target effects resulting from ablation of LRRK2 kinase activity. These might result from impacts that inhibition of LRRK2 play in regulating endosomal–lysosomal pathways (see in the following context). However, recent studies using 3 structural diverse LRRK2 inhibitors at doses that effectively suppress LRRK2 activity, suggest that pathology is mild and reversible after inhibitor is withdrawn [44●●]. Moreover, a further study has reported that one in every ∼500 humans is heterozygous for a loss of function variant in LRRK2, resulting in a ∼50% decrease in LRRK2 protein levels, but this reduction in LRRK2 protein expression apparently has no discernible effect on survival or health [45●]. Available data also suggest that carriers with reduced levels of LRRK2 do not have impaired lung, liver or kidney function [45●]. These results provide firm evidence that therapy leading to a partial reduction of LRRK2 kinase activity should be well tolerated.

## Rab proteins are key physiological LRRK2 substrates

**Figure 2:**
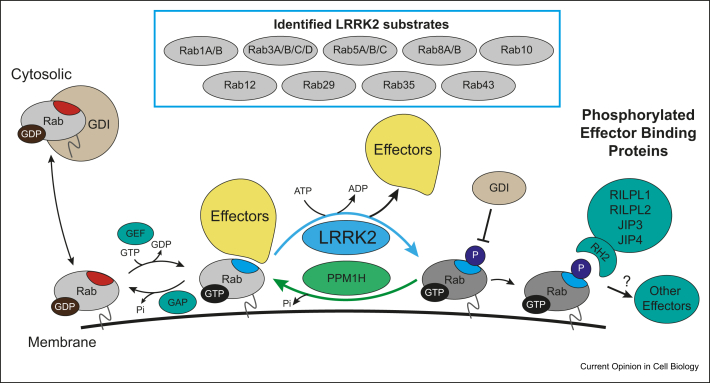
**LRRK2-substrate Rab phosphorylation overview.** Identified LRRK2-substrate Rabs (Rab1A/B, Rab3A/B/C/D, Rab5A/B/C, Rab8A/B, Rab10, Rab12, Rab29, Rab35, Rab43) are phosphorylated at a conserved serine/threonine residue within the Switch-II motif. Rab membrane association is via C-terminal prenylation. Guanine nucleotide exchange factor (GEF) mediated GDP–GTP exchange and activation leading to a conformational change in the Switch-II effector binding motif (red to blue) controls interaction with effectors. GTPase activating proteins (GAP) mediate Rab inactivation via GTP hydrolysis and subsequent recognition by guanine nucleotide dissociation inhibitor (GDI) triggers membrane dissociation to the cytosol. GTP-bearing Rabs interact with effector proteins to elicit downstream functions. LRRK2-mediated Rab phosphorylation leads to dissociation of most Switch-II binding effectors and promotes binding of a set of new phospho-dependent effectors through an RH2-domain [46]. PPM1H mediates selective, LRRK2-substrate Rab dephosphorylation (Rab3A/B/C/D, Rab8A/B, Rab10). RH2, RILP homology domain 2.

The only validated physiological substrates for LRRK2 comprise a subset of Rab proteins including Rab8A and Rab10 [23,46,47] (Figure 2). A number of other LRRK2 substrates have been proposed including endophilin A1 [48], ribosomal protein s15 [49], N-ethylmaleimide sensitive fusion protein [50], synaptojanin [51], P62/SQSTM1 [52] and auxillin [53], but to our knowledge these findings have not been independently confirmed.

A number of Rabs orchestrate membrane trafficking, including the processes of vesicle formation, vesicle movement along actin and tubulin tracks, membrane fusion and regulate synaptic vesicle trafficking [54]. Rabs switch between two conformations, an inactive form bound to GDP and an active form bound to GTP, interacting with effectors through an α-helical Switch-II motif only in the GTP-bound state [55]. Rabs interact with GDP dissociation inhibitors (GDIs), chaperone-like molecules that solubilize and shuttle Rabs between membranes [56]. LRRK2 phosphorylates Rab proteins at a conserved Thr/Ser motif lying at the center of the Switch-II effector binding domain (Figure 2). All the pathogenic LRRK2 mutants enhance Rab phosphorylation *in vivo* consistent with these comprising relevant substrates in PD [23,46,47,57]. Phospho-specific monoclonal antibodies [12●] and sensitive mass spectrometry assays [58●] have been developed to assess endogenous levels of LRRK2 phosphorylated Rab10. In humans, phosphorylation of Rab10 has been assessed in blood-derived neutrophils [59●] and monocytes [60●].

**Figure 3:**
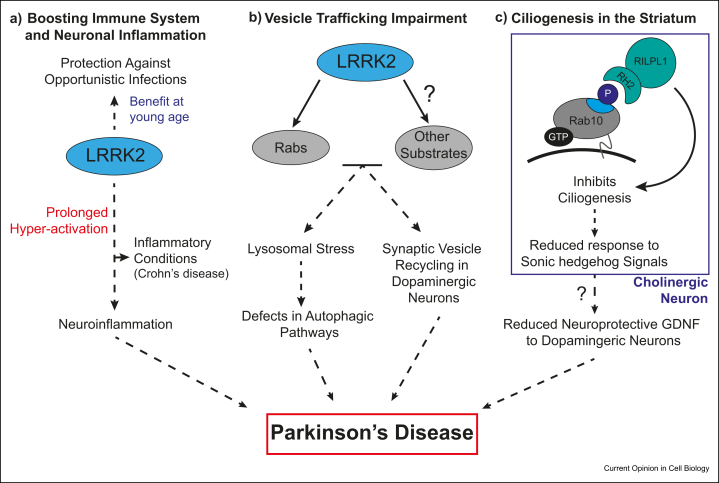
**Proposed mechanisms linking LRRK2 with Parkinson's disease.(a)**. Pathogenic mutations enhancing LRRK2-mediated activity result in boosted immune responses to infectious pathogens. Prolonged hyperactivation may lead to neuroinflammation, increasing PD susceptibility and is linked to chronic inflammatory conditions, such as Crohn's disease. **(b)**. LRRK2 is implicated in vesicular trafficking pathways by phosphorylation of Rabs and other key regulators in the endolysosome system. Dysregulation may lead to (1) lysosomal stress and subsequent defects in autophagic clearance pathways and (2) impaired synaptic vesicle recycling in dopaminergic neurons. **(c)** Elevated LRRK2-mediated activity increases phosphorylated Rab10 and in turn binding of RILPL1 in cholinergic interneurons of the dorsal striatum. This leads to ciliogenesis defects and decreased response to Sonic hedgehog signals from dopaminergic neurons of the substantia nigra. This results in the transmission of less neuroprotective GDNF (glial cell–derived neurotrophic factor) signaling to dopaminergic circuits under stress conditions. Dashed lines indicate a more-than-one step process. RILPL1, Rab interacting lysosomal-like protein 1; LRRK2, leucine-rich repeat protein kinase-2.

LRRK2 is 90% cytosolic and excluded from the nucleus with 10% associating with multiple membrane and vesicle structures [61●●]. The membrane associated LRRK2 has greater specific activity than the cytosolic LRRK2 [11]. LRRK2 associates with and dissociates from distinct membrane compartments where it phosphorylates Rabs [61●●]. Phosphorylation of Rab proteins does not affect GTPase activity but introduces steric clashes preventing association with most Switch-II motif binding effectors including GDI [23,46] (Figure 2). As a result, phosphorylated Rabs become trapped at that membrane location because they cannot be retrieved by GDI [61●●]. A group of effectors that include Rab interacting lysosomal-like protein 1 (RILPL1) and RILPL2 interact with LRRK2 phosphorylated Rab8 and Rab10 [46,62●●,63●]. An RILP homology domain 2 (RH2) in RILPL1 and RILPL2 mediates interaction with phosphorylated Rab8A and Rab10 [46]. The RH2-domain forms a central α-helical dimer that binds to two molecules of pRab8a with the pThr72 binding to a conserved arginine [64●]. RILPL1 and RILPL2 have been implicated in regulating ciliogenesis [65]. PD mutations that activate LRRK2 interfere with ciliogenesis through a pathway involving LRRK2 phosphorylated Rab8A and Rab10 binding RILPL1 [62●●,63●]. In the brain of mice, LRRK2 pathogenic mutations disrupt ciliary signaling in a specific class of cholinergic interneurons of the dorsal striatum, decreasing the ability of these neurons to respond to a Sonic hedgehog signal received from dopaminergic neurons that normally triggers these cells to activate glial cell-derived neurotrophic factor–mediated neuroprotective signaling toward dopaminergic circuits [62●●] (Figure 3). JIP3 and JIP4 scaffolding proteins that have recently been shown to interact with LRRK2 phosphorylated Rab10, also possess an RH2-domain [64●] but the role these proteins play are currently unknown.

At least 20 other Rab proteins are phosphorylated at the equivalent Switch-II motif by kinases other than LRRK2 [46], including Rab7A that is reportedly phosphorylated by LRRK1 promoting its interaction with RILP that also possesses a RH2-domain [66●]. Recently, the PPM1H phosphatase has been shown to dephosphorylate LRRK2 phosphorylated Rabs including Rab8A and Rab10 *in vivo* [67●●]. PPM1H has high catalytic activity and likely plays important roles with other phosphatases to maintain Rab proteins in a dephosphorylated state. It is localized to the Golgi and cytosol and its knockdown suppresses primary cilia formation, similar to pathogenic LRRK2 [67●●].

## Upstream regulators of LRRK2: Rab29 and VPS35

A Golgi-resident Rab protein termed Rab29, encoded within the PARK16 locus mutated in patients with PD [68], has been genetically linked to LRRK2 and PD [69,70]. In overexpression studies, Rab29 interacts with the N-terminal domain of LRRK2 and recruits it to the trans-Golgi network, and greatly stimulates its kinase activity [17●,71●,^72^]. Pathogenic LRRK2 Roc and Cor mutants that promote GTP binding are more readily recruited to the Golgi and activated by Rab29 than wild type LRRK2 [17●]. Rab29 has to be membrane- and GTP-bound to recruit and activate LRRK2. Moreover, LRRK2 phosphorylated Rab10 strikingly accumulates on the membrane where LRRK2 is activated after binding to Rab29, presumably due to phospho-Rab10's inability to bind GDIs [61●●]. LRRK2 also phosphorylates Rab29 at two adjacent residues in the Switch-II motif (Thr71 and Ser72) that may act as a negative feedback loop releasing activated LRRK2 back into the cytosol [17●]. Furthermore, variants within the LRRK2 and Rab29 genes synergistically increase Parkinson's risk [69], and studies in *Caenorhabditis elegans* indicated that RAB29 (GLO-1) ortholog acts upstream of LRRK2 (LRK-1) in a pathway controlling axon termination [73].

A dominant inherited D620N mutation in the gene encoding for VPS35, the cargo binding component of the retromer complex, is associated with late onset PD [74,75]. The retromer complex is made up of 3 proteins (VPS35, VPS29, and VPS26) which package specific endosomal cargoes into vesicles and tubules, and deliver these to either the trans-Golgi network or to the plasma membrane [76]. The VPS35[D620N] mutation markedly elevates LRRK2-mediated phosphorylation of various Rab proteins in fibroblasts and mouse tissues, as well as in human neutrophils and monocytes [60●]. Further studies establish that VPS35 plays a major role in controlling LRRK2 protein kinase activity. Investigations undertaken in mouse and Drosophila concluded that VPS35 and LRRK2 might operate in a common pathway regulating endosomal–lysosomal and Golgi sorting processes [69]. Overexpression of VPS35 ameliorated pathogenic effects on the eye, as well as locomotor deficits and reduced lifespan observed in LRRK2 mutant flies [77]. Drosophila VPS35 and LRRK2 control the same set of synaptic vesicle processes, including dopaminergic synaptic release [78].

## Role of LRRK2 in endolysosomal stress and synaptic vesicle trafficking

LRRK2 has been implicated in endolysosomal stress and impacts on processes orchestrated by the lysosome, such as autophagy and mitophagy [79]. Pathogenic mutations lead to enlarged lysosomes with reduced acidification and degradative capacity, an effect that is reversed by LRRK2 inhibitors [80, 81, 82]. Although LRRK2 and Rabs (Rab8a, Rab10, and Rab29) were reported to be targeted to lysosomes [83] by interacting with the vacuolar-type H + -ATPase Pump a1 subunit to modulate lysosomal pH [84], lysosomal localization of LRRK2 has not been observed in other studies [61●●], and LRRK2 and most Rabs have not been observed in proteomic analysis of purified lysosomes [85]. A recent study reported that a brain-penetrant LRRK2 kinase inhibitor improved endolysosomal and autophagic defects as well as neurodegeneration, caused by exposure of rats to the rotenone toxin [86]. LRRK2 has also been reported to influence synaptic vesicle recycling in dopaminergic and other neurons in many studies [79]. Whether these effects are mediated through phosphorylation of Rabs or other proposed substrates (endophilin A1 [48], N-ethylmaleimide–sensitive fusion protein [50], synaptojanin [51], auxillin [53]) require further analysis (Figure 3). Involvement of Rabs in mediating the impact of LRRK2 in endolysosomal stress and vesicle trafficking could in the future be probed by manipulating expression of the PPM1H phosphatase. Age-dependent dopaminergic neurodegeneration and impairment of the lysosomal pathway has been observed in double LRRK1 and LRRK2 knock-out mice but not in single knock-out animals, suggesting that it would be important that LRRK2 inhibitors being tested do not inhibit LRRK1 [87].

A common risk factor for PD is mutations in the gene GBA1, that encodes for glucocerebrosidase-1, a lysosomal hydrolase that catalyzes the hydrolysis of glucosylceramide to glucose and ceramide [88]. Homozygous loss of function mutations in GBA1 result in a lysosomal storage disorder called Gaucher's disease, but heterozygous loss of function mutations represents a genetic risk factor for PD [88]. A recent study has revealed that pathogenic LRRK2 mutations reduced lysosomal glucocerebrosidase activity in a manner that is reversed by administration of LRRK2 kinase inhibitors in induced pluripotent stem cell–derived dopaminergic neurons [89●]. Mechanistic studies suggest that LRRK2 mediates inhibition of glucocerebrosidase activity via phosphorylation of Rab10 [89●]. These findings suggest that LRRK2 and GBA1 mutations may, in part, contribute to PD pathogenesis through a common mechanism, potentially involving a lysosomal stress pathway.

## LRRK2 in the immune system

LRRK2 is highly expressed in neutrophils, monocytes and macrophages and there is increasing support for LRRK2 playing a role in defense against intracellular pathogens [90,91]. LRRK2 levels in monocytes and B cells are elevated in patients with PD [92,93]. Variants in the LRRK2 gene, such as N2081D located in the kinase domain, enhance Rab10 phosphorylation and have been linked to inflammatory conditions, including Crohn's disease [94●●]. The N2081 is located in the kinase's C-lobe and structural analysis places this in close contact to the LRR region of LRRK2, suggesting a functional relevance of the kinase domain-LRR interface [26●●]. Other work has linked LRRK2 variants, including the gain of function R1628P located in the Cor domain, to be protective against leprosy [95]. Mice lacking LRRK2 activity, or treated with an LRRK2 inhibitor, exhibit impaired ability to clear *S. Typhimurium* infection, whereas G2019S knock-in mice are protected [96,97●]. On the other hand, G2019S mutation augments immune cell chemotaxis and generates more reactive oxygen species during virulent infection [97●]. LRRK2-dependent cellular pathways control *Mycobacterium tuberculosis* replication by regulating phagosome maturation [98●]. If the LRRK2 pathway is hyper-activated over a long period, it could lead to neuroinflammation and increase susceptibility to PD. Consistent with increased inflammation leading to PD, a study reported a 28% increased risk of PD among patients with inflammatory bowel disease [99●●]. Moreover, with anti-inflammatory anti-TNF therapy, PD risk was reduced by up to 78% [99●●]. LRRK2 transgenic mice display more severe colitis induced by dextran sodium sulfate treatment than wild-type mice, which was alleviated by treatment with LRRK2 inhibitors [100●]. The data suggests that in early life, pathogenic mutations that activate LRRK2 could offer protection against opportunistic infection. However, stimulation of proinflammatory responses could increase risk of developing neuroinflammation and PD, especially in later life (Figure 3).

## Potential crosstalk between LRRK2 and alpha-synuclein and tau

A hallmark of sporadic PD is the presence of abnormal protein aggregates termed Lewy bodies within the cytoplasm of neurons, such as the substantia nigra which are most impacted by disease. Alpha-synuclein is the major component of Lewy bodies and consistent with this being a driver of PD, mutations that increase the expression or promote the aggregation of alpha-synuclein are associated with familial forms of PD [101]. Alpha-synuclein is expressed at high levels in the brain and enriched in presynaptic nerve vesicles, but the roles that it plays in regulating synaptic vesicle biology are poorly understood. Alpha-synuclein can form higher order fibril aggregate structures that can propagate through adjacent neurons in the brain. This spread of alpha-synuclein pathology to higher cortical regions correlates with the progression of cognitive decline observed in patients with PD [101]. There has been a lot of interest in whether LRRK2 modulates alpha-synuclein pathology. Several studies have reported that LRRK2 overexpression or G2019S mutation accelerates progression of alpha-synuclein–mediated pathology and aggregation in both primary neurons and mouse models neuropathological changes, whereas deletion of LRRK2 alleviates these alterations [102, 103, •104]. Moreover, the G2019S mutation enhanced alpha-synuclein aggregation, whereas loss of LRRK2 decreased aggregation in human-induced pluripotent stem cell–derived neurons [82,104●].

It has also been reported that antisense oligonucleotides that lower LRRK2 expression in the brain reduced the amount of pathological alpha-synuclein in the substantia nigra of mice inoculated with pathological alpha-synuclein [105]. However, another study aimed at testing the benefit of a brain penetrable LRRK2 inhibitor on a well-characterized overexpression alpha-synuclein mouse model, found that LRRK2 inhibition did not reverse alpha-synuclein pathology, motor phenotypes or neuronal loss [106]. Although the vast majority of patients with sporadic PD display Lewy body pathology autopsy, studies have unexpectedly revealed that Lewy bodies are absent in a significant subset of LRRK2 cases (20 of 37 LRRK2 cases studied and 6 of 17 G2019S PD cases) [107], suggesting that at least in some cases, LRRK2 might drive PD independently from alpha-synuclein or Lewy bodies.

Hyperphosphorylated tau protein accumulates in neurofibrillary tangles in the brains of patients with Alzheimer's disease and, like alpha-synuclein, can be transmitted between neurons. Hyperphosphorylated tau aggregates are commonly observed in sporadic PD. Recent work has shown that tau pathology is a prominent feature of a large majority of LRRK2 mutation carriers [108]. Overexpression [109] or knock-in mutation [110] of pathogenic LRRK2 in mouse brain increases Tau phosphorylation and in future work, it would be interesting to explore whether LRRK2 inhibitors impact Tau pathology and transmission.

The mechanistic link between LRRK2 and alpha-synuclein or Tau is not known. Speculating, LRRK2 could impact aggregation and/or spreading of alpha-synuclein or Tau through phosphorylation of Rab proteins. This could influence endocytosis, autophagy and lysosomal degradation of alpha-synuclein or Tau and hence impact on their steady state levels. Indeed, a recent study has provided evidence that LRRK2 kinase regulates alpha-synuclein spreading in cell culture, nematode, and rodent models via Rab35 phosphorylation [111●]. Recent work has also shown that VPS35[D620N] knock-in mice manifest marked tau neuropathology [112], and it would be interesting to also investigate whether this is impacted with LRRK2 inhibitors.

## Concluding remarks

Researchers have made a reasonable start to deciphering and interrogating the LRRK2 signaling network. Highlights include the discovery that pathogenic mutations activate LRRK2, identification of a subset of Rab proteins as physiological LRRK2 substrates and devising approaches to study pathway activity *in vivo*. The finding that LRRK2 plays pivotal roles in modulating ciliogenesis, the endolysosomal system and immune responses provides insights into how overactivation of LRRK2 is linked to PD. The fact that several companies are in the late stages of preclinical or early phase clinical trials is really exciting. These efforts offer the strongest hopes of developing the first treatments to slow progression or even halt LRRK2-driven PD, which if successful would represent a major landmark in medicine. If these therapies are well tolerated, they could be considered as an approach to prevent PD onset in humans carrying activating LRRK2 or other mutations, such as in VPS35 and perhaps GBA1. Whether such treatments will benefit a subset of patients with idiopathic PD and how to identify those who would respond to LRRK2 inhibitor therapy are major questions that need to be addressed.

Key gaps remain in our knowledge of how LRRK2 is regulated by upstream pathways, such as Rab29, VPS35, the immune system, as well as environmental factors including toxins, herbicides and pesticides, or other PD genes and risk variants. Structural insights into how LRRK2 is regulated and activated, and roles that membrane association, as well as the GTPase domain, microtubule filament and 14-3-3 binding play need to be better defined. Individual functions of the different Rab proteins that are phosphorylated by LRRK2 and the effectors that interact with these after they are phosphorylated need to be determined, as well as how this ultimately impacts downstream biology. It will be important to comprehend what are the most relevant Rab substrates that link LRRK2 to PD using endogenous systems. Our understanding of how LRRK2 regulates the endo-lysosomes is still poorly described and future work should focus on gaining mechanistic insights into this important pathway that is likely key to understanding how LRRK2 is linked to PD.

It should be noted that although the LRRK2 mutant mouse and rat knock-in models have been useful to study the immediate biology that LRRK2 regulates, such as Rab phosphorylation and closely linked downstream biology, these animals do not recapitulate PD. Understanding the reasons for this and developing a faithful mammalian model that develops LRRK2-driven PD would greatly benefit our understanding of how LRRK2 biology is linked to PD.

## Author contributions

M.T. and D.A. worked together to write this review.

## Conflicts of interest statement

Nothing declared.
